# Development and validation of a nomogram to predict cancer-specific survival with unresected cholangiocarcinoma undergoing external radiotherapy

**DOI:** 10.3389/fpubh.2023.1012069

**Published:** 2023-02-02

**Authors:** Jiazhao Song, Yupeng Di, Xiaoli Kang, Gang Ren, Yingjie Wang

**Affiliations:** ^1^Department of Radiotherapy, Air Force Medical Center, PLA, Beijing, China; ^2^Graduate School, Hebei North University, Zhangjiakou, Hebei, China; ^3^Department of Radiotherapy, Peking University Shougang Hospital, Beijing, China

**Keywords:** cholangiocarcinoma, radiotherapy, nomogram, cancer-specific survival, Surveillance, Epidemiology, and End Results (SEER)

## Abstract

**Objective:**

To analyze the prognostic factors of patients with cholangiocarcinoma (CCA) who were unresected and received radiotherapy to establish a nomogram model for the prediction of patient cancer-specific survival (CSS).

**Methods:**

Suitable patient cases were selected from the Surveillance, Epidemiology, and End Results (SEER) database, survival rates were calculated using the Kaplan-Meier method, prognostic factors were analyzed by Lasso, Cox regression, and nomogram was developed based on independent prognostic factors to predict 6 and 12 months CSS. The consistency index (C-index), calibration curve, and decision curve analysis (DCA) were tested for the predictive efficacy of the model, respectively.

**Results:**

The primary site, tumor size, *T*-stage, *M*-stage, and chemotherapy (*P* < 0.05) were identified as independent risk factors after Cox and Lasso regression analysis. Patients in training cohort had a 6 months CSS rates was 68.6 ± 2.6%, a 12-month CSS rates was 49.0 ± 2.8%. The median CSS time of 12.00 months (95% CI: 10.17–13.83 months). The C-index was 0.664 ± 0.039 for the training cohort and 0.645 ± 0.042 for the validation cohort. The nomogram predicted CSS and demonstrated satisfactory and consistent predictive performance in 6 (73.4 vs. 64.9%) and 12 months (72.2 vs. 64.9%), respectively. The external validation calibration plot is shown AUC for 6- and 12-month compared with AJCC stage was (71.2 vs. 63.0%) and (65.9 vs. 59.8%). Meanwhile, the calibration plot of the nomogram for the probability of CSS at 6 and 12 months indicates that the actual and nomogram predict that the CSS remains largely consistent. DCA showed that using a nomogram to predict CSS results in better clinical decisions compared to the AJCC staging system.

**Conclusion:**

A nomogram model based on clinical prognostic characteristics can be used to provide CSS prediction reference for patients with CCA who have not undergone surgery but have received radiotherapy.

## Introduction

Cholangiocarcinoma (CCA) is the most common malignant tumor of the biliary tract, with a high mortality rate despite its rare occurrence, with a survival rate of 5 years only 5–10% (1). CCA has a highly heterogeneous origin within the biliary epithelium and is usually classified into intrahepatic CCA (iCCA), perihilar CCA (pCCA), and distal CCA (dCCA) according to the anatomical site of the secondary bile ducts (2). Current treatment guidelines and consensus recommend R0 radical surgical resection as the treatment modality with the best survival benefit (3, 4). However, these tumors progress insidiously, 70–80% of patients are in the progressive stage at the time of diagnosis, and no more than 30% of patients have the opportunity to undergo radical surgical resection (5, 6). It's difficult to make surgical treatments. Even with the opportunity for surgery, the postoperative positive resection margins rate can still be as high as 64.6–88.2%, with a 5-year survival rate of only 10–30% (6, 7).

For patients with CCA who have lost the chance of resection or refused surgery, as a local treatment, radiotherapy can relieve local symptoms such as pain and obstruction and improve the local control rate while prolonging the survival time for patients with CCA who have lost the chance of resection (8, 9). Although radiotherapy is recommended as a local treatment modality according to the National Comprehensive Cancer Network (NCCN) guidelines, there is a general lack of effective radiotherapy treatment for biliary tract malignancies based on previous studies of the population (10).

In the current era of big data medicine and precision medicine, the use of data models for analysis or prediction is widely used in both the field of oncology and population health assessment (11, 12). In CCA, individualized assessment of cancer survival time and individualized interventions for different patients is particularly important when analyzing the prognostic factors affecting patients with inoperable CCA after radiotherapy due to the individualized differences among patients. The Surveillance, Epidemiology and End Results (SEER) database is characterized by a long time and population-based data, which facilitates prognostic studies in rare diseases. Moreover, as a visual prediction model for comprehensive analysis of prognostic risk factors, the nomogram provides a visual and individualized way to assess prognosis and provides a reference for the clinician's treatment.

To our knowledge, there is a lack of studies on the prognostic assessment of CCA. No nomogram has been developed to assess CSS for patients with unresected CCA receiving radiotherapy. Therefore, this study used a multicenter case collection from the SEER database to analyze and compare the prognostic characteristics of patients with unoperated CCA after radiotherapy, integrate prognostic factors, and create a nomogram to predict the cancer-specific survival (CSS) of patients at 6 and 12 months. The intuitive and simple prediction of nomogram can effectively facilitate the development of precision medicine and provide clinicians with individualized prognosis prediction for different patients, and help improve the quality of communication between physicians and patients with CCA.

## Materials and methods

### Study population selection

The SEER database collects information on cancer patients in 18 Registries in the United States, providing data related to cancer incidence and death for ~30% of the total U.S. population. The authors received approval to access this database (username: 10231-Nov2021). Patients with CCA diagnosed between 2004 and 2015 were identified using the SEER database based on a combination of codes from the International Classification of Diseases in Oncology, 3rd edition (ICD-O-3). The last follow-up was in November 2018. Data from all patients were extracted using SEER^*^Stat software (National Cancer Institute, Bethesda, MD, USA, version 8.4.0). Our reporting followed the Transparent Reporting of Multivariate Predictive Models for Individual Prognosis or Diagnosis guidelines (13). Informed consent and ethical approval did not apply to this study, as all data used were based on publicly available data in the SEER database (https://seer.cancer.gov/data/).

### Data inclusion and exclusion

The primary site codes C22.0, C22.1, and C24.0 were included in the following histology codes 8010, 8020, 8040, 8041, 8070, 8140, 8144, 8160, 8161, 8162, 8163, 8260, 8310, 8480, 8490, or 8560 (14, 15). To ensure the reliability of the data, the exclusion criteria were as follows: patients without a histologically confirmed diagnosis, non-primary tumors, diagnosed only after death or autopsy, unknown cause of death, survival time <1 month after diagnosis, tumors with unknown tumor-node-metastasis (TNM) stage (T0/TX, NX, and MX), unable to receive definitive external radiotherapy, and patients treated surgically at the primary site (RX Summ–Surg Prim Site codes 10, 11, 13, 15–17, 20–27, 30, 36–38, 40, 50–52, 59–61, 65, 66, 75, 90, 99). Variables in data analysis included age at diagnosis, sex, marital status, race, primary site, grade, tumor size, number of primary sites, TNM stage, and chemotherapy. Unresected patients were defined as “RX Summ–Surg Prim Site (1998+)” field codes “00” in the SEER database. The data in the SEER database between 2004 and 2015 correspond to the 6th edition system of TNM. CSS was defined as the duration from the date of diagnosis to the last follow-up or death due to primary cancer causes.

### Building and validating the model

Data from 18 Registries were randomized into training and validation cohorts in a 7:3 ratio, with 13 Registries from the training group and other 5 Registries from the validation group. Cox proportional-hazards models and Lasso regression to assess factors associated with CSS, recording hazard ratio (HR), and 95% confidence interval (CI). Prognostic variables screened by multivariate analysis or meaningful in combination with clinical experience were included in 10-fold cross-validated Lasso regression for further screening to determine independent prognostic factors (*P* < 0.05), and nomogram models were constructed to predict CSS at 6 and 12 months, respectively.

The consistency index (C-index), the area under the curve (AUC), receiver operating characteristic (ROC) curve, and calibration curve were used to test the predictive reliability and predictive ability of the nomogram, respectively. C-index > 0.5 and calibration curve distribution close to the diagonal of the prediction model indicated good predictive performance. Internal validation was performed by the Bootstraps method with 1,000 resamples and external validation using a validation cohort to further evaluate the model applicability. The clinical usability of the nomogram was estimated using decision curve analysis (DCA).

### Statistical analysis

Categorical variables were expressed as numbers and percentages (%), and continuous variables were expressed as Mean (SD). Categorical variables tests were performed by χ^2^ test or Fisher's exact test. Continuous variables were compared by *t*-test or *u*-test. Survival analysis was performed using the Kaplan-Meier method. All analyses were performed with R v.4.4.1 statistical software (http://www.R-project.org, The R Foundation, Vienna, Austria), and SPSS 25.0 (IBM Corporation, Armonk, NY, USA) was used for comprehensive statistical analysis of the collected data. A two-tailed *P* < 0.05 was considered statistically significant.

## Results

### Population baseline characteristics

**Figure 1:**
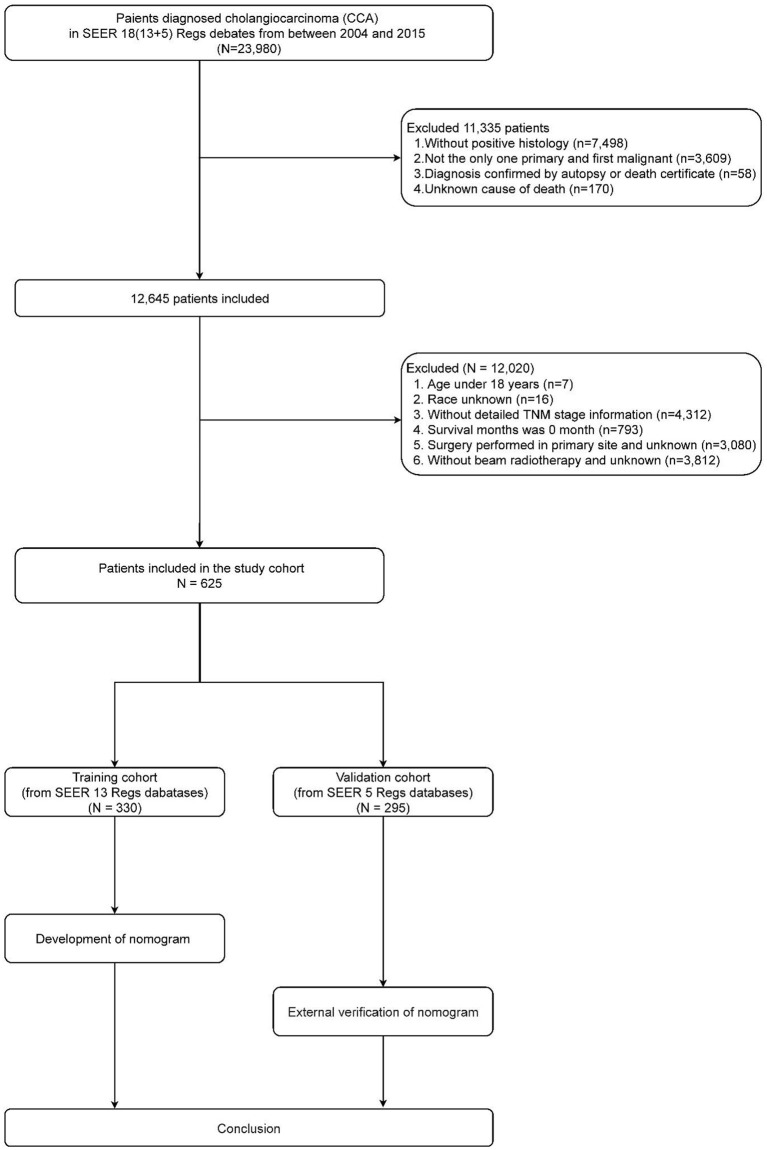
Flow chart for cholangiocarcinoma patients.

A total of 625 CCA patients were selected for analysis. The training cohort consisted of 330 patients for nomogram construction and the validation group of 295 patients. The detailed process of patient selection is summarized in Figure 1. The differences in basic information between the training and validation group were not significant, and only two variables, race and primary site of the tumor, were statistically significant (*P* < 0.05).

Patient characteristics in the training set were as follows: Mean (SD) age was 66.1 ± 12.1 years, white race 71.8% (237/330), 172 (52.1%) were male gender, and more than half of the participants were married (62.4%, 206/330), and 75.5% of the patients had received chemotherapy. In addition, the tumors were mostly primary in the intrahepatic bile duct (47.0%), with similar numbers of patients with moderately differentiated (16.7%) and poorly differentiated (17.3%) tumors. Most patients had tumors larger than 50 mm (109/330, 33.0%). Detailed information is shown in Table 1.

**Table 1:** Baseline characteristics of patients in the training cohort and validation cohort from SEER database.

**Characteristic**	**Total (*n* = 625)**	**Training (*n* = 330)**	**Validation (*n* = 295)**	***P*-value**
Age				0.265
<65	302 (48.3)	152 (46.1)	150 (50.8)	
≥65	323 (51.7)	178 (53.9)	145 (49.2)	
Age, Mean (SD)	65.6 (53.6, 77.6)	66.1 (54.0, 78.2)	65.0 (53.1, 76.9)	0.211
Sex				0.653
Male	332 (53.1)	172 (52.1)	160 (54.2)	
Female	293 (46.9)	158 (47.9)	135 (45.8)	
Marital status				0.377
Married	386 (61.8)	206 (62.4)	180 (61.0)	
Single	75 (12.0)	45 (13.6)	30 (10.2)	
Others^*^	142 (22.7)	69 (20.9)	73 (24.7)	
Unknown	22 (3.5)	10 (3.0)	12 (4.1)	
Race				<0.001
White	478 (76.5)	237 (71.8)	241 (81.7)	
Black	50 (8.0)	24 (7.3)	26 (8.8)	
Others^**^	97 (15.5)	69 (20.9)	28 (9.5)	
Primary site				<0.001
Liver	104 (16.6)	49 (14.8)	55 (18.6)	
Intrahepatic bile duct	250 (40.0)	155 (47.0)	95 (32.2)	
Extrahepatic bile duct	271 (43.4)	126 (38.2)	145 (49.2)	
Grade				0.605
I	27 (4.3)	13 (3.9)	14 (4.7)	
II	103 (16.5)	55 (16.7)	48 (16.3)	
III	104 (16.6)	57 (17.3)	47 (15.9)	
IV	3 (0.5)	3 (0.9)	0 (0.0)	
Unknown	388 (62.1)	202 (61.2)	186 (63.1)	
Tumor size				0.195
<30 mm	102 (16.3)	52 (15.8)	50 (16.9)	
30–50 mm	107 (17.1)	65 (19.7)	42 (14.2)	
>50 mm	201 (32.2)	109 (33.0)	92 (31.2)	
Unknown	215 (34.4)	104 (31.5)	111 (37.6)	
*T* stage				0.687
T1	215 (34.4)	107 (32.4)	108 (36.6)	
T2	79 (12.6)	45 (13.6)	34 (11.5)	
T3	211 (33.8)	113 (34.2)	98 (33.2)	
T4	120 (19.2)	65 (19.7)	55 (18.6)	
*N* stage				0.647
N0	443 (70.9)	237 (71.8)	206 (69.8)	
N1	182 (29.1)	93 (28.2)	89 (30.2)	
*M* stage				0.513
M0	416 (66.6)	224 (67.9)	192 (65.1)	
M1	209 (33.4)	106 (32.1)	103 (34.9)	
Chemotherapy				0.461
No/unknown	162 (25.9)	81 (24.5)	81 (27.5)	
Yes	463 (74.1)	249 (75.5)	214 (72.5)	

* Divorced, separated, and widowed;

** American Indian/AK Native and Asian/Pacific Islander.

### Analysis of prognostic factors

**Figure 2:**
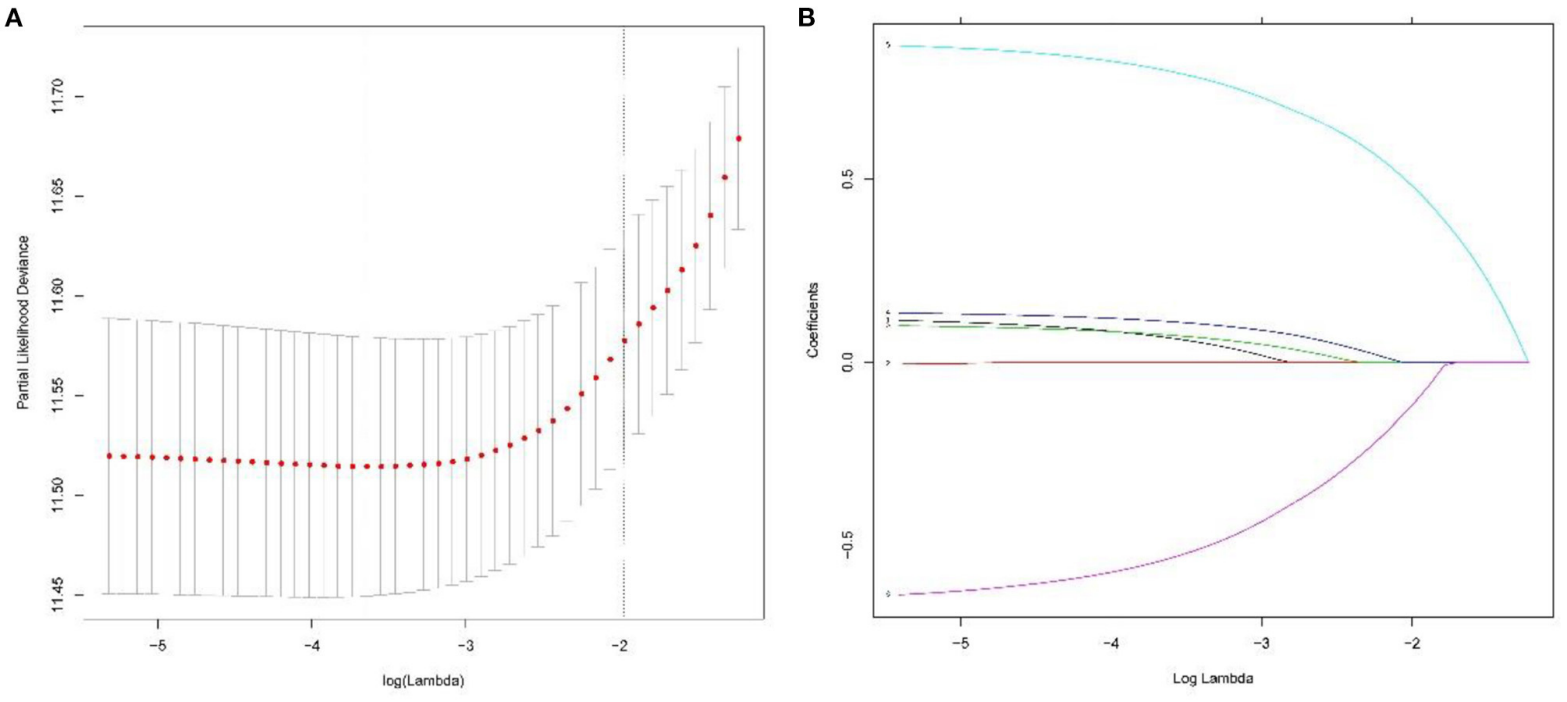
To further narrow the range of variables involved in the regression analysis, the parameters were adjusted by 10-fold cross-validation, and established using the least absolute shrinkage and selection operator (LASSO) in the Cox model in the training set **(A)**. Combining the distribution of LASSO coefficients for six variables (tumor primary site, grade, tumor size, *T* stage, *M* stage, and chemotherapy) in cholangiocarcinoma patients, an optimal lambda filter was used to generate five variables (tumor primary site, tumor size, *T* stage, *M* stage, and chemotherapy) with non-zero coefficients **(B)**.

As shown in Figure 2, the median CSS time for the training set was 12.00 months (95% CI: 10.17–13.83 months), with a 6-month CSS of 68.6 ± 2.6% and a 12-month CSS of 49.0 ± 2.8%. Multivariate Cox regression analysis showed that patients with the primary site in the extrahepatic bile duct, the tumor size over 30 mm, T4 stage, M1 stage, and without chemotherapy had a poor cancer-specific prognosis (HR > 1, *P* < 0.05) (Table 2). Combined with clinical experience, grades of differentiation were included in Lasso regression for further screening (Figure 1). Ultimately, according to Table 2, multivariate proportional-hazards regression and Lasso regression analysis identified four variables (tumor primary site, tumor size, *T* stage, *M* stage, and chemotherapy) as independent prognostic factors (*P* < 0.05).

**Table 2:** Multivariate analyses for CSS in patients with CCA.

**Characteristic**	**Multivariate**	
	**HR (95% CI)**	***P*** **-value**
**Age**		
<65	Ref	
≥65	1.05 (0.82~1.34)	0.706
**Sex**		
Male	Ref	
Female	0.96 (0.75~1.23)	0.744
**Marital status**		
Married	Ref	
Single	0.88 (0.61~1.26)	0.484
Others^*^	1.35 (0.98~1.86)	0.065
Unknown	0.66 (0.32~1.34)	0.252
**Race**		
White	Ref	
Black	1.55 (0.96~2.49)	0.071
Others^**^	0.75 (0.55~1.02)	0.069
**Primary site**		
Liver	Ref	
Intrahepatic bile duct	1.22 (0.85~1.74)	0.274
Extrahepatic bile duct	1.62 (1.07~2.44)	0.022
**Grade**		
I	Ref	
II	1.11 (0.58~2.14)	0.746
III	1.17 (0.61~2.24)	0.643
IV	0.59 (0.13~2.76)	0.507
Unknown	1.07 (0.59~1.94)	0.824
**Tumor size**		
<30 mm	Ref	
30–50 mm	1.59 (1.07~2.38)	0.022
>50 mm	1.58 (1.06~2.35)	0.024
Unknown	1.54 (1.06~2.23)	0.023
***T*** **stage**		
T1	Ref	
T2	1.25 (0.85~1.83)	0.258
T3	1.21 (0.88~1.67)	0.231
T4	1.52 (1.07~2.17)	0.020
***N*** **stage**		
N0	Ref	
N1	1.10 (0.84~1.43)	0.490
***M*** **stage**		
M0	Ref	
M1	2.80 (2.11~3.71)	<0.001
**Chemotherapy**		
No/Unknown	Ref	
Yes	0.46 (0.34~0.63)	<0.001

* Divorced, separated, and widowed;

** American Indian/AK Native and Asian/Pacific Islander.

CSS, cancer-specific survival; CCA, cholangiocarcinoma.

### Nomogram construction and validation

**Figure 3:**
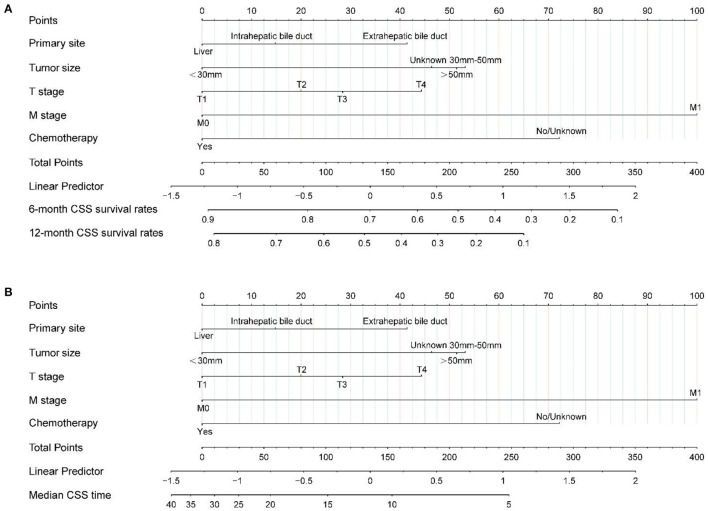
Nomogram **(A, B)** predicting 6-, 12-month, and median CSS of patients with radiotherapy in unresected cholangiocarcinoma based on 5 prognostic factors. CSS, cancer-specific survival.

Based on the independent prognostic factors identified after the above-mentioned multifactorial regression, nomogram were constructed, and the corresponding scores for each variable were specified by combining each patient's characteristics. Finally, the 6 and 12 months CSS were estimated based on the total score after summing the variables to assess the probability of survival for different individuals, as detailed in Figure 3.

**Figure 4:**
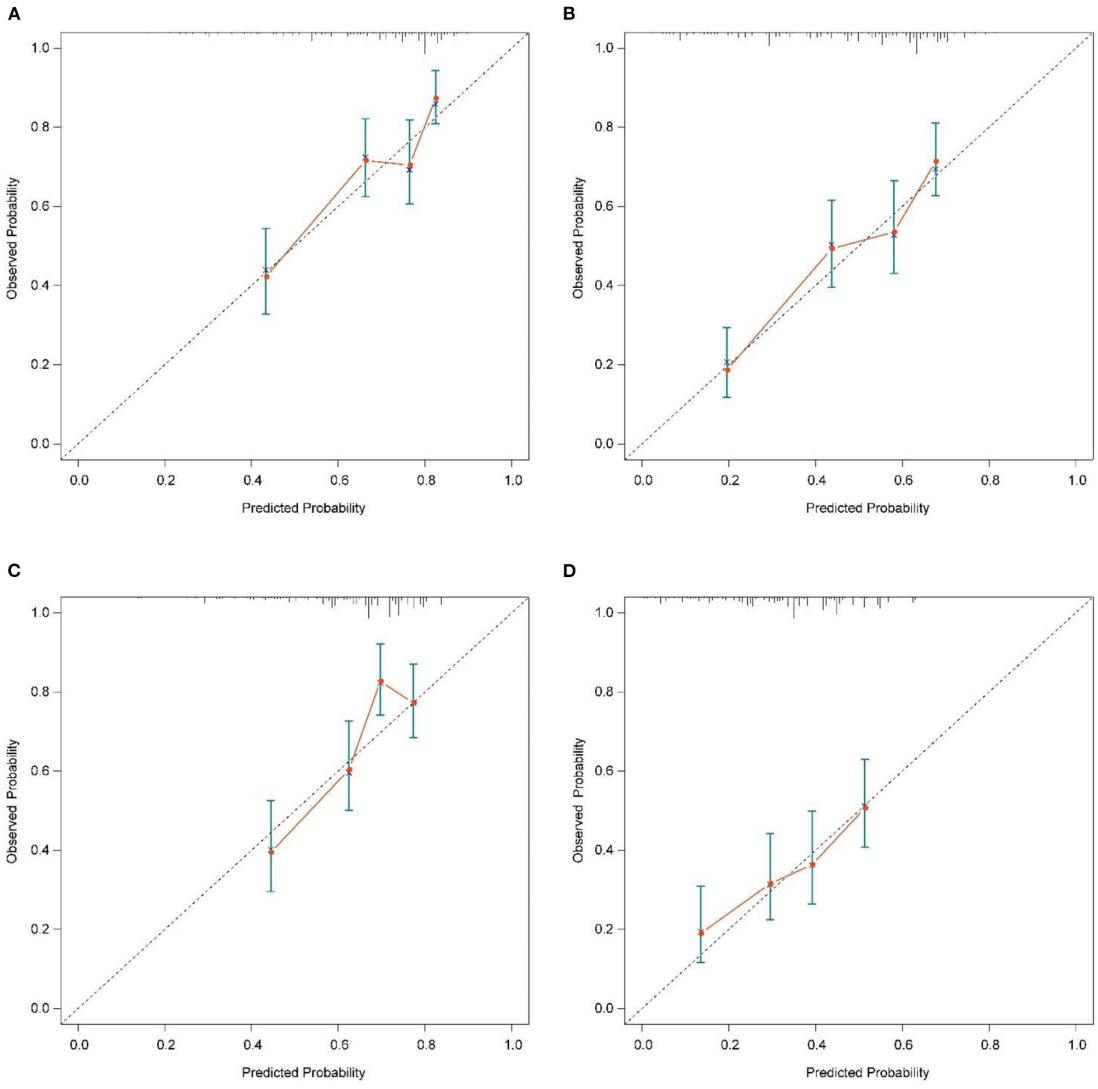
Calibration plots of the nomogram for 6-, and 12-month CSS prediction of the training set **(A, B)** and verification set **(C, D)**. X-axis represents the nomogram-predicted probability of survival; Y-axis represents the actual CSS probability. A perfectly accurate nomogram prediction model would result in a plot that the observed and predicted probabilities for given groups fall along the 45-degree line. Dots with bars represent nomogram-predicted probabilities along with 95% confidence interval. CSS, cancer-specific survival.

**Figure 5:**
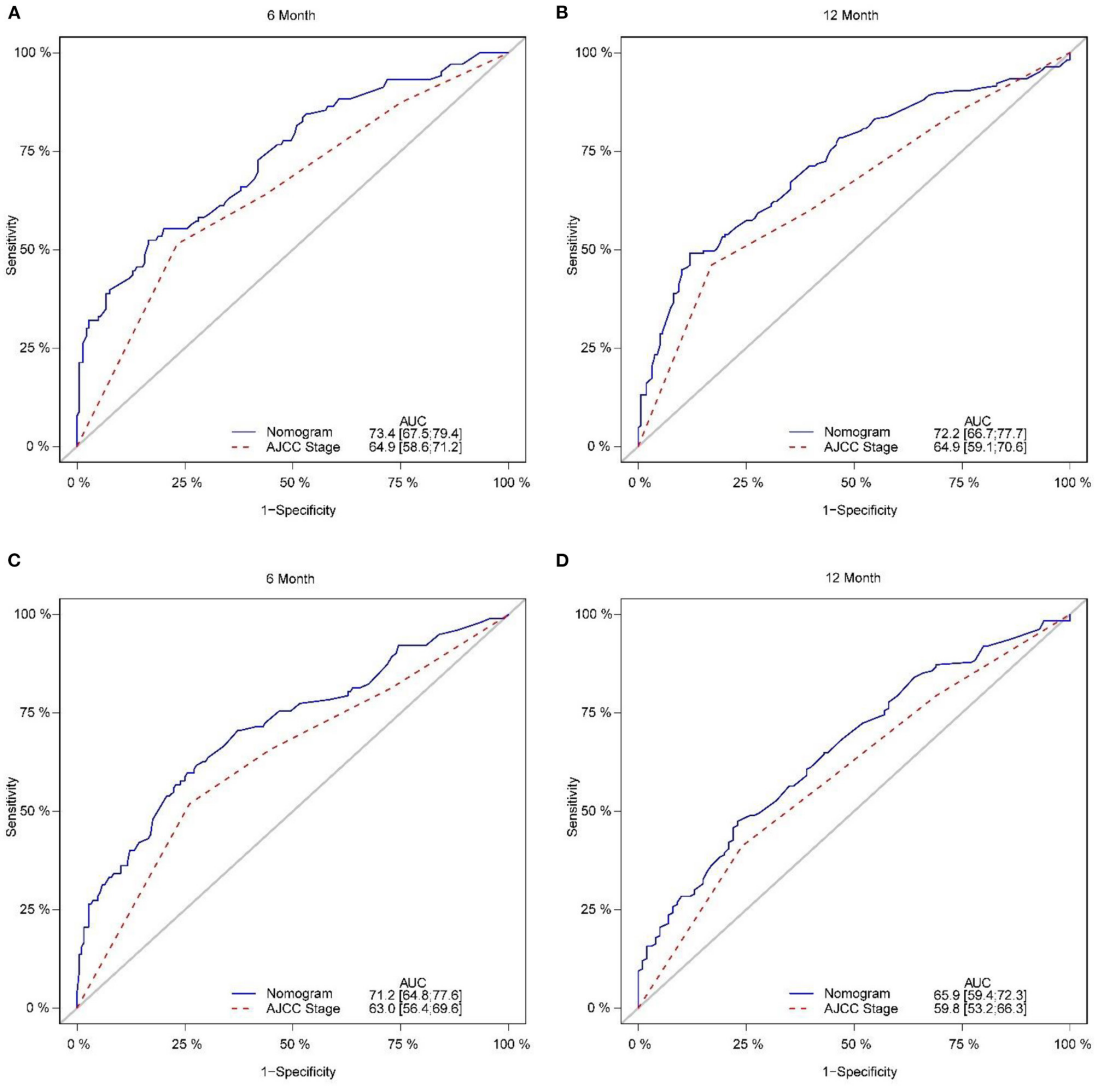
Comparison of the ROC curves of the nomogram and the TNM stage system for 6-, and 12-month CSS prediction in the training set **(A, B)**. And the ROC curves of the nomogram for 6-, and 12-month CSS prediction in the verification set **(C, D)**. CSS, cancer-specific survival.

The calibration plot of the nomogram for the probability of CSS at 6 and 12 months showed agreement on whether the training set or validation set (Figure 4), which indicates that the actual and nomogram predict that CSS remains largely consistent at 6 and 12 months. C-index was 0.664 (95% CI, 0.625–0.703) for the training set and 0.645 (95% CI, 0.603–0.688) for the validation set. The graphs ROC curves were compared of the nomogram with AJCC stage for 6 and 12 months and the corresponding AUC for 6 (73.4 vs. 64.9%) and 12 months (72.2 vs. 64.9%), respectively (Figures 5A, B).

**Figure 6:**
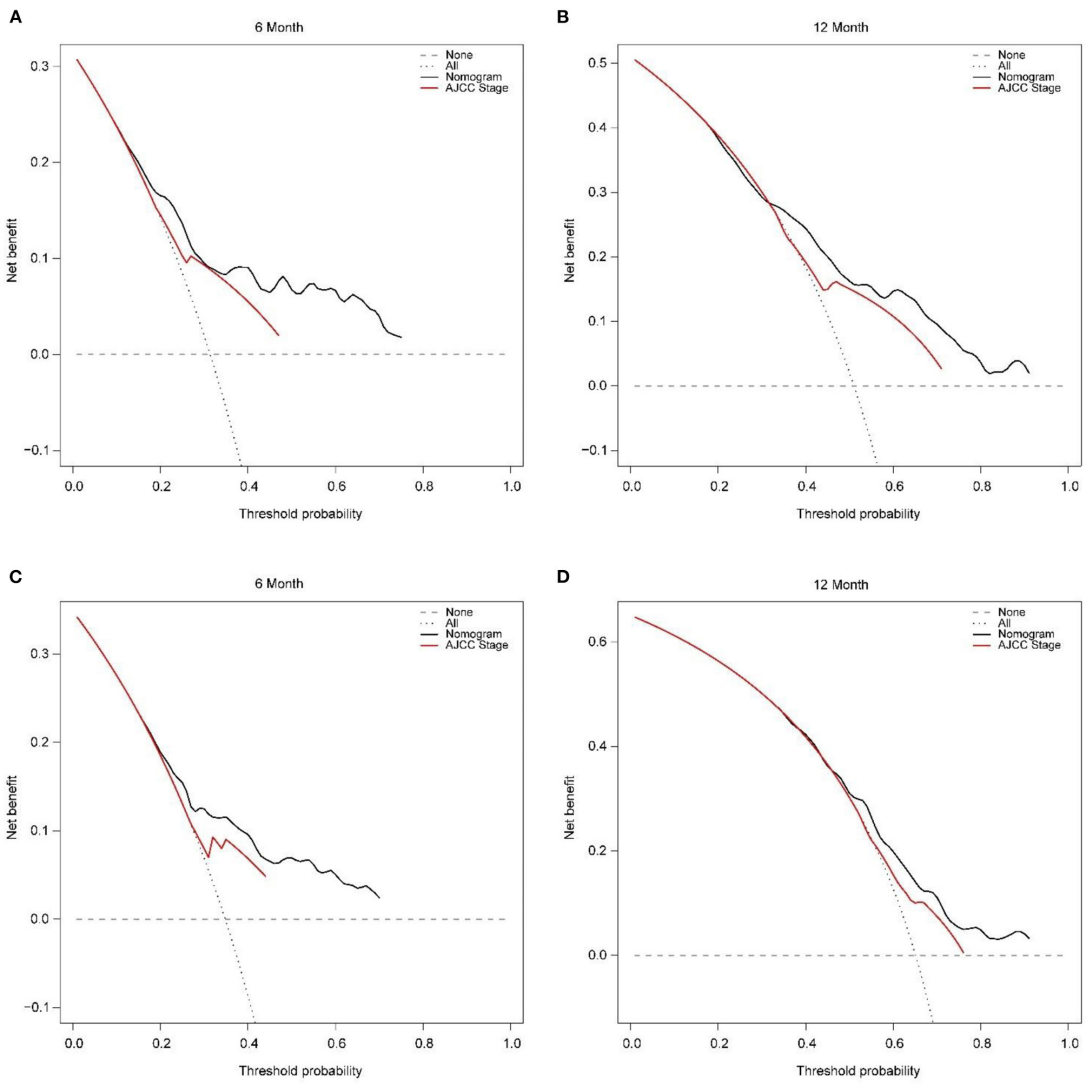
Decision curve analysis of training set **(A, B)** and validation set **(C, D)** compare with the AJCC stage for predicting 6 months CSS and 12 months CSS. CSS, cancer-specific survival.

In addition, external validation calibration plot is shown in Figures 5C, D, AUC for 6- and 12-month compared with AJCC stage was (71.2 vs. 63.0%) and (65.9 vs. 59.8%). DCA showed that using a nomogram to predict CSS results in better clinical decisions compared to the AJCC staging system (Figure 6).

### Clinical application of nomogram

Each patient can calculate a total score based on the scores of individual variables in Figure 3, and then obtain the corresponding 6 and 12-month predicted survival rates on the nomogram based on the total score. For instance, a patient with a primary tumor size of 30–50 mm in the extrahepatic bile duct, T2, M0 stage has not received surgical treatment only received radiotherapy and chemotherapy. Combined with the legend, the total points were about 88. Corresponding to 6 and 12 months CSS was about 78–80% and 60–65%, respectively. The median CSS time was about 15–17 months. It is recommended that patients with unresectable CCA receive radiotherapy to prolong the CSS.

## Discussion

Radiotherapy is an effective treatment modality for patients with inoperable CCA with poor prognoses (9, 16, 17). In this study, we analyzed the prognostic factors affecting patients with CCA who received radiotherapy but did not undergo surgery based on the SEER database, and we found poor prognosis for patients with tumor primary site in the extrahepatic bile duct, tumor size larger than 30 mm, M1 stage, and not combined with chemotherapy. Previous prognostic studies of patients with CCA based on large databases have focused on the perioperative period. Most previous studies of prognostic factors in patients with CCA have focused on three aspects: (1) preoperative prediction of intraoperative or postoperative outcomes (18, 19); (2) postoperative assessment of surgical outcomes (20–22), and (3) evaluation of perioperative prognosis based on imaging histology (23–26). To our knowledge, this is the first study to create a nomogram to assess the CSS for patients with unresected CCA treated with radiotherapy. While identifying prognostic factors, the predictive accuracy and predictive validity of the nomogram were evaluated comprehensively to prevent overfitting of the prediction model and to solve clinical decision problems using DCA, making the developed and constructed nomogram more practical.

There are multiple reasons why CCA cannot be resected, mostly at the patient level, due to the difficulty of tolerating surgery at an advanced age or psychological rejection of invasive surgery, and at the tumor level, due to the presence of vascular invasion and late staging, making it difficult to achieve surgical cure. In conjunction with the present study, more than half of the patients were older than 65 years and more than 30% were T4. In a population-based treatment modality and prognosis study, it was shown that for inoperable elderly patients with iCCA, receiving radiotherapy significantly improved overall survival compared to no treatment (27). This confirms that radiotherapy is a viable non-invasive treatment for patients with unresectable CCA. Among them, tumor size and *m*-staging can be present as independent prognostic factors, which is consistent with the results of this study. However, this study expanded the population distribution based on the elderly iCCA, but at the same time limited the treatment modality of the included population and only analyzed the prognosis of radiotherapy for non-operated patients. Combined with other prognostic studies in CCA, married young female patients may have a better prognosis (27–29). However, in the present study, sex, age, marital status, and race were not prognostic factors, a result that reflects the heterogeneity of results caused by the different included populations and suggests the need for individualized assessment of prognosis.

It has been shown that pathologic grading is a predictor of survival in patients with operable CCA (30). However, in a study of inoperable CCA, our study found that pathologic grade was not an independent prognostic factor. Tumor size > 3 cm may indicate a poor prognosis, consistent with the findings of inoperable patients, and is an important factor in CCA prognosis (17, 31, 32). In addition, distant metastasis is a major cause of poor prognosis in patients with CCA in the late stages, and survival is usually difficult beyond 0.5 years (8).

In this study, ~76% of patients received radiotherapy in combination with chemotherapy, and these patients had a more significant survival benefit than radiotherapy alone, which is consistent with the results of a previous study on prognostic factors for radiotherapy (33). In recent years, there has been interested in combining radiotherapy with chemotherapy in patients with CCA. Relevant retrospective studies have shown that chemoradiotherapy is superior to radiotherapy or chemotherapy alone in terms of tolerability, side effects, local control rate, PFS, or OS (34–36). The rapid development of targeted therapy or immunotherapy has become a new and effective treatment method to improve the survival rate of patients with CCA. The treatment mode of high-dose radiotherapy combined with immunity is more significant than that of traditional radiotherapy in terms of the effect of activating tumor immune response (37). Clinical experience suggests that stereotactic radiation therapy combined with immune checkpoint inhibitors can give iCCA patients who are initially inoperable to have the opportunity to undergo surgery (38). Studies have shown that for patients with pCCA and dCCA, changes in the immune status of tumor tissue after radiotherapy are related to the effect of treatment, and patients with higher lymphocyte expression levels of PD-L1 and CD8+ cytotoxins in tumor tissues before and after combined chemoradiotherapy have a poor prognosis (39). In summary, immune checkpoint inhibitors or targeted drugs combined with radiotherapy for CCA are new treatment options.

Earlier studies have revealed CCA-specific prognostic variables. For example, carbohydrate antigen 19-9 (CA19-9) levels are a commonly used biomarker to study the prognosis of biliary malignancies (40, 41). The CA19-9 expression can be an independent risk factor for prognosis in patients with CCA, and elevated CA19-9 suggests poor prognosis. However, there are still some patients with CCA who have normal CA19-9 expression (42). Platelet-to-lymphocyte ratio (PLR) and albumin (ALB) can be independent predictors of OS preoperatively (43). Although the above variables can help predict prognosis, reports of prognostic factors for OS in CCA remain variable across studies of different populations. Compared to established prognostic factors, the prediction of nomograms may provide a more individualized assessment of patient prognosis.

In one of the few studies on radiotherapy nomogram prediction, Song et al. (44). developed a column line graph model combining age, gender, tumor site, histological differentiation, neural infiltration, and lymph node metastasis to predict OS and recurrence-free survival (RFS) in patients with pCCA and dCCA treated with postoperative adjuvant radiotherapy through single-institution retrospective analysis. Some previous studies published in the SEER database, which analyzed prognostic factors and predicted OS based on different CCA staging, generated a higher C-index than AJCC staging and confirmed the accuracy of columnar maps over the AJCC staging system through validation (29, 30, 45). However, there is still a lack of prognostic models for radiotherapy in patients with unoperated CCA. Our study fills this gap by building a training set with a C-index of 0.653 ± 0.037 and a validation set with a C-index of 0.626 ± 0.040, respectively, based on the previous study and combining data information from the SEER database, by constructing nomograms to predict OS at 0.5 and 1 year. Considering that the primary site is associated with CCA staging and survival and may affect the prognosis clinical application of the model.

Admittedly, there are still several limitations of the present study that may make our nomogram C index and AUC values not relatively high. First, because the SEER database does not provide detailed information about radiotherapy and chemotherapy, some positive prognostic variables such as the degree of vascular invasion, liver function, degree of biliary obstruction, and some important tumor markers, such as CA19-9, are likewise absent in the SEER dataset. Perhaps the addition of these variables could improve the predictive power. Second, the reasons why patients did not undergo surgery are not clear, and some patients for non-oncologic reasons, such as family financial reasons or comorbid severe organic disease, may have a selection bias, and we still need to conduct further studies on prospective cohorts in the future.

## Conclusions

In conclusion, we found that primary site, tumor size, T stage, M stage, and chemotherapy were independent risk factors for CSS in patients with CCA who had received radiotherapy but did not undergo surgery. Our population-based and above risk factors study was the first to establish a nomogram to predict CSS for patients with CCA who were unresected and received radiotherapy. This nomogram has shown good accuracy and clinical applicability through internal validation and external validation. It shows better potential application than conventional TNM staging and can help clinicians make clinical decisions. Providing an individualized prognostic reference for patients with unresected CCA undergoing radiotherapy.

## Data availability statement

Publicly available datasets were analyzed in this study. This data can be found here: https://seer.cancer.gov/.

## Author contributions

YW conceived and designed the study. JS, YD, XK, and GR collected data and analyzed the literature. JS, YD, and XK analyzed and interpreted the data. JS drafted the manuscript. All authors contributed to the critical revision, editing, and approval of the final manuscript.
